# Assessement of Survival and its Affecting Factors in Adult Liver Transplant Patients: A Retrospective Cohort Study

**DOI:** 10.34172/aim.34367

**Published:** 2026-01-01

**Authors:** Malihe Safari, Javad Faradmal, Sina Mohagheghi, Zohreh Khajehahmadi, Saman Nikeghbalian, Ghodratollah Roshanaei

**Affiliations:** ^1^Department of Biostatistics, School of Medicine, Arak University of Medical Sciences, Arak, Iran; ^2^Clinical Research Development Unit of Amiralmomenin Hospital, Arak University of Medical Sciences, Arak, Iran; ^3^Department of Biostatistics, School of Public Health, Modeling of Noncommunicable Diseases Research Canter, Hamadan University of Medical Sciences, Hamadan, Iran; ^4^Department of Clinical Biochemistry, Faculty of Medicine, Hamadan University of Medical Sciences, Hamadan, Iran; ^5^Shiraz Organ Transplant Center, Shiraz University of Medical Sciences, Shiraz, Iran; ^6^Clinical Research Development Unit of Valiasr Hospital, Arak University of Medical Sciences, Arak, Iran

**Keywords:** Adult patient, Cox model, Liver transplantation, Survival analysis

## Abstract

**Background:**

Liver disease is a leading cause of mortality among adults worldwide. Liver transplantation (LT) remains the only definitive treatment for patients with end-stage liver failure and has shown considerable success, particularly in high-volume centers in developing countries. Numerous factors can influence long-term survival following LT. This study aimed to identify the factors associated with survival among adult liver transplant recipients at Namazi Hospital, Shiraz between 2001 and 2018.

**Methods:**

This retrospective cohort study included 3712 adult patients who underwent liver transplantation for advanced liver failure. Demographic and clinical data were extracted from medical records. Cox regression models were used to assess factors associated with post-transplant survival. Data was analyzed using the SPSS and R software.

**Results:**

Of the 3712 patients, 742 (20%) died during follow-up. Also, 2348 (63.3%) patients were male, and the mean (SD) age was 42.3 (13.2) years (range: 19–74 years). In the multivariable Cox model, re-transplantation, older recipient and donor age, higher Model for End-Stage Liver Disease (MELD) score, and certain etiologies of liver disease were significantly associated with poorer survival. Conversely, transplantation performed in 2010 or later was independently associated with improved survival outcomes.

**Conclusion:**

Older recipient, donor age, and higher MELD score were independently associated with higher mortality after liver transplantation. Patients transplanted from 2010 onward experienced better survival, reflecting advancements in transplant care over time. Additionally, compared to acute liver failure (ALF), etiologies such as primary sclerosing cholangitis (PSC), autoimmune hepatitis (AIH), Budd-Chiari syndrome, cryptogenic liver disease, hepatitis B virus (HBV), and primary biliary cholangitis (PBC) were associated with significantly lower mortality risk and improved long-term survival.

## Introduction

 Liver transplantation is the most appropriate treatment for advanced liver failure.^1^ The first successful liver transplantation in the world in an animal model was performed in 1955 and the first human liver transplant was reported in 1968 in the United States.^2^ The first liver transplant in Iran was performed in 1993 in Shiraz Namazi Hospital.^3^ Although treatment progress has improved patient survival, liver transplantation remains the only effective option for end-stage liver failure, and outcomes depend on multiple factors.^4^

 According to the European Liver Transplant Registry datathe 1-, 10- and 18-year survival rates in LT were 83%‒88%, 68%‒72% and 48% – 55% respectively, while in Europe in 2020, the 1- and 5- year survival rates were 86% and 74%.^6-7^ Based on a meta-analysis of 117 liver transplant survival studies, 1-, 2-, 3-, 5- and 10-year survival rates were estimated to be 85%, 80%, 75%, 73% and 71%, respectively.^8^ The 1-, 3- and 5-year survival rates in Iran have been reported to be 85%, 82% and 79%, respectively.^9^

 Various studies have been conducted to evaluate the effect of transplant recipients and donors’ characteristics on the survival of patients and each of them has identified different factors affecting the patients’ survival.^10-18^

 Considering the importance of studying the factors affecting the success of liver transplantation in adults and also considering that there is no study with this number of adult samples in Iran, the aim of this study was to determine the effect of donor and recipient characteristics on the survival of liver transplant patients in Iran.

## Materials and Methods

 In this retrospective cohort study, we evaluated adult liver transplant patients over 18 years of age. Required information including donor and recipient characteristics were extracted from the patients’ records. The survival status of patients was ascertained using periodic visits and telephone calls. Before liver transplantation (LT), cirrhosis and its underlying causes were diagnosed through clinical symptoms, imaging, and laboratory tests, with confirmation via histopathological examination of the explanted liver. Cirrhosis etiologies in more than 45 cases were classified as primary causes, including acute liver failure (ALF), alcoholism, autoimmune hepatitis (AIH), Budd-Chiari syndrome, hepatitis B and C infections, nonalcoholic steatohepatitis (NASH), primary biliary cholangitis (PBC), primary sclerosing cholangitis (PSC), and Wilson’s disease. Patients with multiple contributing factors were categorized under ‘mixed etiology,’ while rarer conditions were grouped as ‘other.’ This structured classification enhances our understanding of the various conditions leading to cirrhosis in transplant candidates.^19^

 The proportional hazards assumption of Cox model was tested using Schoenfeld residuals test. Model diagnostics and assessment of goodness-of-fit were conducted using the concordance index, likelihood ratio test, and score test. The overall survival of the patients from transplantation to death or end of the follow-up period was calculated in terms of months. Kaplan-Meier and proportional hazard cox models were used to estimate survival and determine the affecting factors. All obtained data were analyzed using IBM SPSS, version 23 and R software (version 4.4.3). Statistical significance was set at *P* < 0.05.

## Results

 A total of 3712 adult liver transplant patients were included in the analysis, of whom 2348 (63.3%) were male. The mean (SD) age of recipients was 42.3 (13.2) years (range: 19 to 74 years). The mean (SD) age of donors was 36.1 (15.2) years (range: 9 to 76 years). A total of 742 (20%) patients died by the end of follow-up. Table 1 highlights several demographic and clinical factors significantly associated with outcomes in liver transplant recipients. Transplantation year (after 2010), recipient and donor age, recipient and donor BMI, MELD score ( > 20), etiology of liver disease, donor blood group, re-transplantation status, and ICU admission were all significantly associated with survival (*P* < 0.01). In contrast, recipient sex, donor sex, donor-recipient sex pairing, graft type, recipient blood group, and blood group compatibility showed no statistically significant association with outcomes.

**Table 1:** Demographic and clinical characteristics of donors and recipients

**Variable**	**Subgroup**	**N (%)**	**Number of death**	***P*** ** value**
Year of Transplantation	< = 2010	726(19.6)	243(33.5)	**<0.01**
	> 2010	2986(80.4)	499(16.7)	
Recipient Sex	Female	1364(36.7)	276(20.2)	0.79
	Male	2348 (63.3)	466(19.8)	
Recipient Age	19‒39	1614 (43.5)	286(17.7)	**<0.01**
	40-59	1730 (46.6)	350(20.2)	
	≥ 60	368 (9.9)	106(28.8)	
Donor Sex	Female	1080(29.1)	241(22.3)	0.52
	Male	2632(70.9)	501(19)	
Donor Age	< 20	580(15.6)	89(15.3)	**<0.01**
	20‒39	1595(43)	302(18.9)	
	40‒59	1274(34.3)	279(21.9)	
	≥ 60	263(7.1)	72(27.4)	
Paired Sex	F→F	432(11.6)	96(22.2)	0.13
	M→F	932(25.1)	180(19.3)	
	F→M	648(17.5)	145(22.4)	
	M→M	1700(45.8)	321(18.9)	
Recipient BMI	< 18.5	183(4.9)	27(14.8)	**<0.01**
	18.5‒25	1535(41.4)	237(15.4)	
	> 25	1081(29.1)	154(14.2)	
	unknown	913(24.6)	324(35.5)	
Donor BMI	< 18.5	50(1.3)	7(14)	**0.01**
	18.5‒25	892(24)	124(13.9)	
	> 25	676(18.2)	111(16.4)	
	unknown	2094(56.4)	500(23.9)	
MELD	< = 20	1669(52.2)	220(13.2)	**<0.01**
	> 20	1526(47.8)	522(25.6)	
Etiology of Liver Disease	HBV	792(21.3)	175(22.1)	**<0.01**
	Cryptogenic	645(17.4)	117(18.1)	
	PSC	642(17.3)	96(15)	
	AIH	511(13.8)	94(18.4)	
	Wilson	168(4.5)	28(16.7)	
	NASH	147(4)	34(23.1)	
	HCV	125(3.4)	37(29.6)	
	Budd-Chiari	119(3.2)	22(18.5)	
	Mixed	117(3.2)	20(17.1)	
	HCC	73(2)	25(34.2)	
	ALF	59(1.6)	18(30.5)	
	PBC	55(1.5)	7(12.7)	
	Alcoholic	48(1.3)	9(18.8)	
	Other*	216(5.8)	60(27.8)	
Graft Type	partial	111(3)	23(20.7)	0.81
	Whole Organ	3601(97)	719(20)	
Recipient Blood Group	A	1117(30.1)	222(19.9)	0.36
	B	921(24.8)	170(18.5)	
	AB	307(8.3)	58(18.9)	
	O	1367(36.8)	292(21.4)	
Donor Blood Group	A	1089(29.3)	205(18.8)	**0.01**
	B	887(23.9)	155(17.5)	
	AB	276(7.4)	47(17)	
	O	1460(39.3)	335(22.9)	
Number of Transplantation	First transplantation	3623(97.6)	695(19.2)	**<0.01**
	Retransplantation	89(2.4)	47(52.8)	
ICU Addmission	Yes	2734(73.7)	600(21.9)	**<0.01**
	No	978(26.3)	142(14.5)	
Blood Group	matched	3594(96.8)	709(19.7)	0.69
	Mildly mismatched	49(1.3)	13(26.5)	
	strongly mismatched	26(0.7)	5(19.2)	
	Bidirectional matched	43(1.2)	9(20.9)	

* The “other” category of etiologies, along with cases of unknown cause, account for a smaller share of liver transplant cases compared to the more prevalent causes.

 The mean (SD) MELD score was 21.4 (6.7). Furthermore, 3396 (99.5%) of the patients received a transplant from a dead donor. Table 1 presents the characteristics of donors and recipients by the survival status.

**Figure 1:**
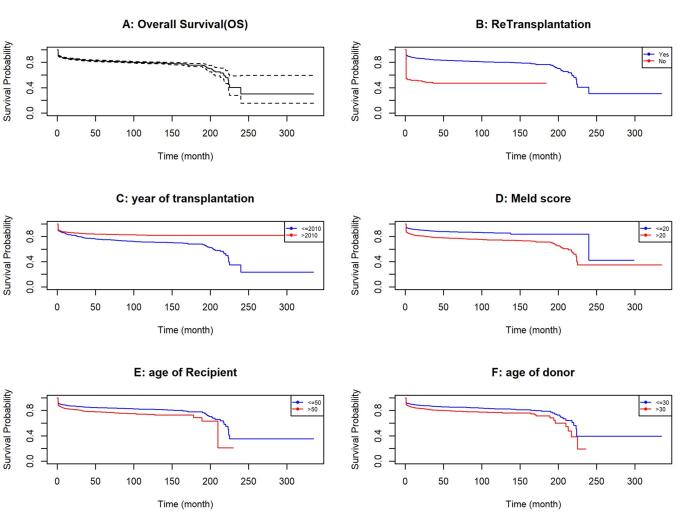
(A) Overall survival for all patients (B) Overall survival by re-transplantation status (yes vs. no). (C) Comparison of survival based on the year of transplantation (≤ 2010 vs. > 2010), reflecting potential changes in transplant practices or policies over time. (D) Survival stratified by Model for End-Stage Liver Disease (MELD) score at the time of transplantation (≤ 20 vs. > 20), indicating disease severity. (E) Impact of recipient age on post-transplant survival, categorized as ≤ 50 years vs. > 50 years. (F) Effect of donor age on recipient survival, stratified as ≤ 30 years vs. > 30 years

 A Kaplan-Meier model was used to estimate the survival of patients, with the results showing the overall survival probability (OS) of the patients (Figure 1A).

 The mean (SD) and median follow-up durations were 74.6 (49.3) and 70 months, respectively. The median (IQR) survival time was 224 (186, 246) months. Moreover, the 1-, 3-, 5-, 10-, 15-, and 20-year survival rates were 86%, 83%, 82%, 79%, 76% and 61%. respectively.

 To evaluate the proportional hazards (PH) assumption, we analyzed Schoenfeld residuals. The assumption appears to be reasonably met for all covariates, as none of the individual tests were statistically significant (all *P* > 0.05). Additionally, the global test supports the overall validity of the PH assumption (χ^2^ = 42.605, *P* = 0.22).

 According to standard model evaluation criteria, the Cox proportional hazards model demonstrates an excellent fit to the data. This is evidenced by strong overall statistical significance across all three global tests—the Likelihood Ratio, Wald, and Score tests. The model’s C-index of 0.715 reflects good discriminatory ability, indicating that it effectively differentiates between patients at higher and lower risk of death. These findings suggest that the model is not only statistically sound but also potentially clinically useful, provided the included variables are relevant and interpretable. Notably, the Likelihood Ratio test yielded χ^2^ = 136.3 with 33 degrees of freedom (*P* < 0.001), further confirming the model’s robustness and significance.

 To evaluate the impact of potential risk factors on patient survival, both univariable and multivariable Cox regression analyses were performed. In the univariable analysis, transplant year, recipient age, donor age, MELD score, disease etiology, blood group, ICU admission, and re-transplantation were all significantly associated with survival (*P* < 0.05) (Table 2).

**Table 2:** Multivariable Cox Regression model of Prognostic Factors on OS

**Variable**	**Subgroup**	**Unadjusted HR (95% CI)**	***P*** **-Value**	**Adjusted HR (95% CI)**	***P*** **-Value**
Recipient Age		1.02(1.01‒1.05)	0.01	1.02(1.01‒1.03)	0.01
Donor age		1.01(1.008‒1.02)	0.04	1.008(1.001‒1.015)	0.04
MELD		1.04(10.03-1.06)	0.01	1.05(1.04‒1.06)	0.02
Year of Transplantation	< = 2010	1		1	
	> 2010	0.62(0.53‒0.73)	< 0.01	0.61(0.47‒0.77)	< 0.01
Paired gender	F→F	1			
	M→F	0.82(0.64‒1.05)	0.75		
	F→M	0.98(0.76‒-1.28)	0.58		
	M→M	0.81(0.65‒1.02)	0.35		
Recipient BMI	18.5‒25	1			
	< 18.5	0.92(0.62‒1.37)	0.62		
	> 25	1.22(0.94‒1.58)	0.58		
Etiology of liver disease	ALF	1		1	
	PSC	1.73(1.12‒2.71)	0.01	0.41(0.24‒0.73)	< 0.01
	Wilson	0.69(0.56‒0.86)	< 0.01	0.42(0.31‒1.03)	0.75
	HCC	0.73(0.51-1.05)	0.11	1.09(0.58‒1.93)	0.65
	Mixed	1.83(1.25‒2.67)	0.02	0.49 (0.26‒1.06)	0.51
	Other	0.84(0.55‒1.28)	0.66	0.87(0.51-1.47)	0.48
	Alcoholic	1.51(1.16‒1.94)	0.04	0.51(0.22-1.11)	0.39
	AIH	0.86(0.47‒1.59)	0.63	0.47(0.28‒0.78)	0.02
	Budd-Chiari	0.80(0.65‒0.99)	0.03	0.52(0.26‒0.97)	0.03
	Cryptogenic	0.91(0.62-1.34)	0.76	0.46(0.22‒0.76)	0.01
	HBV	0.81(0.66‒0.99)	0.04	0.59(0.36‒0.95)	0.03
	HCV	1.01(0.85‒1.21)	0.52	0.81(0.42‒1.42)	0.68
	NASH	1.43(1.04‒1.960	0.04	0.73(0.41‒1.29)	0.73
	PBC	1.26(0.91‒1.76)	0.34	0.28(0.11‒0.71)	0.02
Graft type	Partial& Split	1			
	Whole Organ	0.95(0.64‒1.43)	0.89		
ICU admission	No	1		1	
	Yes	0.79(0.65‒0.95)	0.01	0.81(0.65‒1.01)	0.21
Retransplantation	No	1		1	
	Yes	3.79(2.82‒5.11)	< 0.01	4.15(2.62‒6.61)	< 0.01
Blood Group	Matched	1			
	Mildly mismatched	1.313(0.76‒2.27)	0.73	1.01(0.52‒1.99)	0.43
	Strongly mismatched	1.23(1.11‒2.39)	0.04	1.38(0.57‒3.33)	0.28
	Bidirectional matched	1.27(0.46‒1.72)	0.84	1.43(0.71‒2.92)	0.59

**Figure 2:**
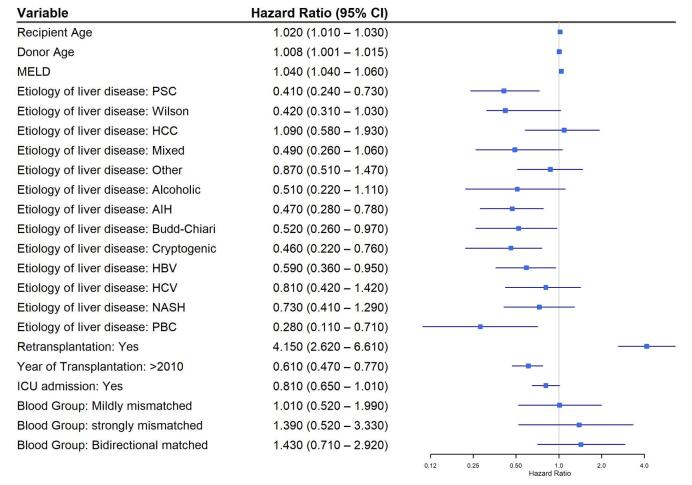
Forest Plot of Adjusted Hazard Ratios for Mortality After Liver Transplantation

 The Kaplan–Meier (KM) survival curves for the key prognostic variables identified in the multivariable Cox proportional hazards model are displayed in Figure 1 (B-F). These curves depict the estimated survival probabilities over time across categories of each significant variable, highlighting differences in survival outcomes. To complement these survival curves, a forest plot of the multivariable hazard ratios (HRs) with corresponding 95% confidence intervals is provided, offering a comprehensive visual summary of the relative effect sizes and enhancing the interpretability of the model results. (Figure 2).

## Discussion

 This study examined long-term survival among adult patients with end-stage liver disease in Iran and evaluated the influence of donor and recipient characteristics on post-transplant outcomes.

 In this study, recipient age was significantly associated with survival; younger recipients had better outcomes, consistent with prior research. Similar findings were reported by Madreseh *et al*. (9), Song *et al*.,^10^ and Pischke *et al.*,^15^ all of whom observed that increasing recipient age was linked to higher mortality risk and that younger patients experienced improved survival. Gómez-Gavara *et al*. further noted that recipients aged over 65 (particularly those with alcohol-related liver disease) had markedly reduced survival rates.

 Donor age also emerged as a critical factor in our analysis: older donor age was associated with increased recipient mortality.^16^ This aligns with a meta-analysis by Mohan,^17^ which found that recipients of livers from donors aged ≥ 70 years had significantly lower 1- and 5-year survival compared to those receiving grafts from younger donors. Filali *et al.*^18^ reported that donor age > 53 years and recipient age > 36 years were both linked to higher mortality risk. Similarly, Zhou *et al*.,^20^ Northup *et al.*,^11^ Heinemann *et al.*,^21^ and Pozo-Laderas *et al*.^12^ consistently demonstrated that advanced age in either donors or recipients, particularly beyond thresholds of 50–60 years, was associated with poorer post-transplant survival. Collectively, these findings underscore age as a pivotal determinant of liver transplant outcomes. This could be because the effect of aging has an effect on the function of different organs and decreases organ function; so, using younger donors increases the chances of survival in recipients, and therefore transplant recipients have a better chance of survival at the age lower than 50.

 In the present study, the most common etiologies of liver disease among liver transplant (LT) recipients were hepatitis B virus (HBV), cryptogenic cirrhosis, primary sclerosing cholangitis (PSC), and autoimmune hepatitis (AIH). In contrast, Wong *et al.* reported that in the United States, the leading causes of liver disease among LT recipients particularly those without hepatocellular carcinoma (HCC) were non-alcoholic steatohepatitis (NASH) and alcohol-related liver disease (ALD), with NASH increasingly becoming a dominant indication for transplantation.^22^ Our findings indicate that patients transplanted for HBV had a significantly lower risk of death. This is consistent with Pischke *et al*., who identified HBV as the only etiology independently associated with improved patient survival compared to other underlying liver diseases.^15^ Conversely, Filali *et al.* found that HCV and other etiologies were linked to significantly higher mortality,^18^ while Madreseh *et al.* reported that autoimmune hepatitis (AIH) and HCC more than doubled the risk of death, and other causes increased mortality by more than fourfold.^9^ Kollmann *et al. *observed no significant association between disease etiology and survival among patients aged over 65 years,^23^ and Zhou *et al.* reported that, using HCV as the reference, only HBV was associated with reduced mortality, with other etiologies showing no significant impact on survival.^20^

 Low risk of death in HBV-positive patients in this study may be due to the fact that all HBV-positive liver transplant recipients received drugs to prevent transplant reinfection.

 The results of this study showed that the year of transplantation was statistically significantly associated with the risk of death. The risk of death in transplant recipients decreased significantly in the last decade compared to the previous decade, so that the risk of death in patients receiving transplantation after 2010 decreased by approximately 44% and this decrease was statistically significant. In the study by Yoon *et al*., the risk of death after 2010 was significantly reduced.^24^ In the study by Filali *et al.*, the risk of death was significantly reduced after 2000.^18^ In the study by Kollmann et al., conducted in patients over 65 years of age, the risk of death in patients transplanted after 2005 increased by almost two-fold in the univariable model, but year of transplant was not significant in the multivariable model.^23^ In the study by Pommergaard *et al.*, the year of transplant did not have a significant effect on the risk of death.^25^

 This observation may be explained by advances in surgical techniques and post-transplant care, such as access to more effective and less complex drugs, the development of less invasive procedures, and the growing experience of transplant teams in surgery, anesthesia, and post-transplant management over the past decade.

 In line with other studies, high MELD score increased the risk of death in patients. In the study by Pommergaard *et al*., MELD score had a significant effect on the risk of death.^25^ Using a machine learning method, Lankerani *et al.* showed Mold score to be the most important factor affecting the survival of adult transplant patients.^26^ Also, Northup *et al.*,^11^ Pozo-Laderas *et al*.^12^ and Zhou *et al*.,^20^ demonstrated that high MELD score had a negative effect on survival, but Kollmann *et al.* showed that MELD score did not affect survival in patients over 65 years of age.^23^

 Patients with higher MELD scores are often in more advanced stages of liver disease, which complicates their clinical management and increases the likelihood of complications. This can lead to a vicious cycle where the worsening condition further elevates the MELD score, thereby threatening survival.

 In the current study, re-transplantation was performed in only 2.4% of patients—a rate consistent with reports from the United States (2–3%)^27^ and Asia (~3%),^28-29^ but lower than those in Europe (6.6%)^30^ and Australia (6.7%).^13^ Despite its life-saving potential in select cases, re-transplantation was associated with a more than threefold increase in mortality risk. This finding is supported by Northup *et al*., who reported a more than twofold higher risk of death among re-transplanted patients.^11^ A meta-analysis by Brombosz *et al.* further identified key predictors of poor outcomes after re-transplantation, including advanced recipient age, higher MELD score, and elevated serum creatinine—factors that significantly impact both patient and graft survival. Additionally, recipients of livers from older donors faced a higher risk of post-re-transplant mortality.^31^ In contrast, Croome *et al.* found no significant difference in survival between patients undergoing primary versus repeat transplantation,^32^ underscoring variability across centers and populations. Overall, although re-transplantation remains a critical rescue option, it is linked to substantially higher mortality compared to primary liver transplantation, likely due to limited donor availability, greater recipient frailty, and complications such as graft failure, vascular thrombosis, and infection.

 In this study, mild, strong, and bidirectional blood group mismatches were associated with an increased risk of mortality, and this increase was statistically significant in the univariable analysis (*P*< 0.05). However, after adjusting for other variables in the multivariable model, the effect of blood group compatibility on survival was no longer significant. The results of the study by Yoon *et al*. on adult patients with hepatocellular carcinoma who received transplants from living donor, showed that the survival rate of patients did not have any significant difference between the ABO-incompatible and ABO-compatible groups.^14^ In the study by Yang *et al.*, the risk of death in univariable and multivariable models in ABO-incompatible patients was 4.16 and 2.8 times, respectively. They assessed survival rates at different levels of the MELD score and concluded that for recipients with MELD scores ≤ 30, receiving an ABO-incompatible liver transplant had a prognosis comparable to ABO-compatible. For recipients with MELD scores ≥ 40, ABO-incompatible transplantation should be undertaken with caution.^33^ Also, in the studies by Zhou *et al*.^34^ and Zhang *et al*.,^35^ blood group mismatch had no significant effect on survival. However, while ABO-incompatible liver transplantation is possible, it carries a higher risk of rejection, poorer survival rates, and increased complications compared to ABO-matched or compatible transplants.

 The observed association between ICU admission and reduced mortality in the univariable model appears counterintuitive, as ICU admission typically reflects greater illness severity and higher clinical acuity. Although this association did not remain statistically significant in the multivariable model, it warrants cautious interpretation due to potential confounding or selection bias. One possible explanation is reverse causation; patients who deteriorate rapidly and die before ICU admission are excluded from the ICU group, potentially creating an artificial survival advantage among those who are admitted. Alternatively, differences in ICU admission criteria, timing of transfer, center-specific management protocols, or unmeasured confounders may contribute to this finding. Notably, while ICU admission was significantly associated with survival in the univariable analysis, this effect was attenuated after adjusting for other covariates, suggesting that its apparent protective association may be mediated or confounded by factors such as disease severity, comorbidities, or transplant-related complications. This unexpected result should be interpreted with caution and merits further investigation.

 The strengths of this study include its emphasis on long-term survival, which provides more valuable insights into post-transplant care and management compared to studies focusing solely on short-term outcomes. Additionally, the large sample size enhances the reliability of the findings. The use of advanced statistical techniques, such as Cox proportional hazards models, enables robust multivariable analysis and adjustment for potential confounding factors.

 However, the study has several limitations. Its retrospective design may lead to missing or incomplete data and limits causal inference, as it can only identify associations rather than establish causality. Additionally, temporal changes in medical practices, surgical techniques, immunosuppressive regimens, and donor selection criteria may not be fully captured when evaluating the impact of transplantation year. Finally, re-transplantation occurred in only 2.4% of patients, lower than the rates reported in international studies which may limit the statistical power to draw robust conclusions about outcomes following re-transplantation.

## Conclusion

 In conclusion, recipient age, donor age, MELD score, re-transplantation, transplant era (post-2010), and underlying liver disease etiology emerged as independent predictors of survival following liver transplantation. Advanced recipient and donor age, higher MELD scores, and re-transplantation were associated with significantly increased mortality risk. In contrast, transplantation performed after 2010 was linked to improved survival, likely reflecting advances in surgical techniques, perioperative care, and immunosuppression. Moreover, patients transplanted for PSC, PBC, AIH, HBV, Budd-Chiari syndrome, or cryptogenic liver disease exhibited better long-term survival compared to those with acute liver failure (ALF). These findings underscore the critical role of judicious patient and donor selection, as well as the positive impact of evolving transplant practices, in optimizing post-transplant outcomes.
